# Supplementary material for: Effectiveness of COVID-19 Vaccines over 13 Months Covering the Period of the Emergence of the Omicron Variant in the Swedish Population
Source: Vaccines (Basel). 2022 Dec 5;10(12):2074. doi: 10.3390/vaccines10122074 (PMC9782222; doi:10.3390/vaccines10122074)
Supplement: Supplementary file 1 [file vaccines-10-02074-s001.zip › vaccines-2065268-supplementary.pdf]

Supplemental Material

Table of Contents

Section S1: Data cleaning of vaccine data obtained from National Vaccine Register (NVR) 3

Section S2: Data Source of covariates and definition of prior comorbidities and treatments 3

Section S3: Statistical Analysis 3

Example of data structure to estimate time-varying vaccine effectiveness 3

Time intervals used in the statistical analysis for each COVID-19 outcome 4

Modelling steps in 10% random samples 4

Analysis of vaccine effectiveness for first dose 5

Section S4: Results and Discussion on vaccine effectiveness for first dose 5

Table S1. Comparison between models without and with adjusting covariates, in 10% randomly selected individuals. Analyses were performed for COVID-19 infection, estimating vaccine effectiveness (VE) after two doses. 6

Table S2. Analysis performed in dose 1 and dose 2 separately and analysis including two doses simultaneously with an interaction term between dose and time interval. Analyses were performed for COVID-19 infection in 10% randomly selected individuals. 7

Table S3. Vaccine effectiveness (VE) against COVID-19 infection and hospitalization per time interval after one dose 9

Table S4. Vaccine effectiveness (VE) against COVID-19 ICU admission and death per time interval after one dose 10

Table S5. Intervals between vaccine doses among individuals who received at least two doses of vaccination. 11

Table S6. Vaccine effectiveness (VE) against COVID-19 infection and hospitalization per time interval after two doses 12

Table S7. Vaccine effectiveness (VE) against COVID-19 ICU admission and death per time interval after two doses 13

Table S8. Vaccine effectiveness (VE) against COVID-19 infection during pre-Omicron and Omicron period, per time interval after two doses 14

Table S9. Vaccine effectiveness (VE) against COVID-19 hospitalization during pre-Omicron and Omicron period, per time interval after two doses 15

Figure S1. Trend of COVID-19 infection (a) and hospitalization (b) from January 1, 2020 to January 31, 2022 16

Figure S2. Cumulative proportion of vaccine uptake over time among whole Swedish population (a) and 65+ (b) 17

Figure S3. Cumulative count of homologous vaccine type over time 18

Figure S4. Vaccine effectiveness against COVID-19 infection (a), hospitalization (b) and severe outcomes [ICU admission (c), death (d)] after one dose. 19

Figure S5. Vaccine effectiveness against COVID-19 infection (a, c) and hospitalization (b, d) after two doses in each age group 20

Figure S6. Overall vaccine effectiveness against COVID-19 infection (a) and hospitalization (b) after two doses in each sex stratum 21

Figure S7. Overall vaccine effectiveness against COVID-19 infection (a) and hospitalization (b) by different homologous vaccine type among individuals above age 65. 22

References:........ 23

Section S1: Data cleaning of vaccine data obtained from National Vaccine Register (NVR)

Vaccination data, the date of vaccination and the brand of vaccine for each dose, was obtained from the National Vaccination Register (NVR), at the Public Health Agency of Sweden. A very short time gap between two doses is likely to represent errors in the data or erroneous repeat registrations of the same dose. Therefore, the second and the following doses were defined as the next vaccination record that is not shorter than the "recommended" minimum time gap from the first dose. The length of the required minimum time gap was decided according to the guidelines for COVID-19 vaccination and depended on the type of vaccine received at the earlier dose. We used 19 days for BNT162b2, and 25 days for AZD1222 and mRNA-1273. The guidelines for BNT162b2 were that the second dose should be 3-7 weeks from the first dose, and it was eventually set to 19 days in order to allow some flexibility around the guidelines. Similarly, for AZD1222 and mRNA-1273, it was recommended to be 4-7 weeks, and was set to be 25 days.

Section S2: Data Source of covariates and definition of prior comorbidities and treatments

This study is part of the larger SCIFI-PEARL project with regularly updated data from various National Registers (1). SCIFI-PEARL retrieved sociodemographic data including country of birth, education, family situation, income and occupational data from 2018 from the Longitudinal Integrated Database for Health Insurance and Labour Market Studies (LISA) from Statistics Sweden (SCB). Information on elderly subjects living at special care facilities and/or receiving home care services came from the National Social Service Register.

Additionally, SCIFI-PEARL retrieved a complete specialist care medical history for all individuals from the Swedish National Patient Register (NPR) from 2015, and a complete drug history for prescription drugs from the National Prescribed Drug Register (NPDR) from 2019. The five-year prior medical history (using ICD-10 codes) and one-year prior prescription drugs history (using ATC codes) were used to define the prior comorbidities and treatments.

**Prior comorbidities and treatments**	**Description**	**ICD-10 or ATC codes**	**ATC codes**
Cardiovascular diseases	Diagnosis from NPR	I05-I09, I20-I51, R001, R011, Q20-Q28	NA
Stroke	Diagnosis from NPR	I60-I62, I630-I635, I638-I639, I64	NA
Hypertension	Diagnosis from NPR, or on medication for hypertension from NPDR	I10-I15	C02-09
Diabetes (type 1 and type 2)	Diagnosis from NPR, or on medication for diabetes from NPDR	E10, E11	A10A, A10B
Obstructive respiratory disease	Diagnosis of COPD/asthma from NPR, or on medication for obstructive airway diseases from NPDR	J44, J45	R03A, R03BA, R03BB, R03C, R03D
chronic kidney diseases	Diagnosis from NPR	N17-N19	NA
Obesity	Diagnosis from NPR	E66	NA
Autoimmune diseases	Diagnosis from NPR	M05-M14	NA
Dementia	Diagnosis from NPR	F00-F03	NA
Psychiatric conditions	Diagnosis from NPR, or on medication for neuroleptics, anxiolytics, sedatives or anti-depressants from NPDR	F20-F39	N05A, N05B, N05C, N06A
Cancer	Diagnosis from NPR	C00-C97	NA

Section S3: Statistical Analysis

Example of data structure to estimate time-varying vaccine effectiveness

This study aimed to estimate time-varying vaccine effectiveness (VE), meaning different VE estimates during different certain time intervals after vaccination. To achieve that, in the Cox model, each individual's follow-up time was first divided according to vaccination status (unvaccinated, first dose and

second dose) and then the vaccination exposure periods were further divided into time intervals after each dose until transition to the next dose.

Below is an example of data structure. For the first study participant (ID=1), the person-time was divided into 7 records, corresponding to one period under unvaccinated status and six periods under first dose, and censored when infection happened during week 7-8 after first dose. For the second study participants (ID=80), the person-time was divided into 8 records, corresponding to one period under unvaccinated status and four periods under first dose and three periods under second dose. Note that the time interval for this person was not continuous but interrupted by vaccination status changing. This person was censored when infection happened during week 3 after second dose. The third study participant (ID=96) only had one record as he was infected during the unvaccinated period.

**Study ID**	**enter**	**out**	**Vaccination status**	**Time interval**	**Infection date**	**event**
1	1 Jan 2020	23 Feb 2021	unvaccinated	0	13 Apr 2021	0
1	23 Feb 2021	2 Mar 2021	dose 1	Week 1	13 Apr 2021	0
1	2 Mar 2021	9 Mar 2021	dose 1	Week 2	13 Apr 2021	0
1	9 Mar 2021	16 Mar 2021	dose 1	Week 3	13 Apr 2021	0
1	16 Mar 2021	23 Mar 2021	dose 1	Week 4	13 Apr 2021	0
1	23 Mar 2021	6 Apr 2021	dose 1	Week 5-6	13 Apr 2021	0
1	6 Apr 2021	13 Apr 2021	dose 1	Week 7-8	13 Apr 2021	1
…						
80	1 Jan 2029	16 Mar 2021	unvaccinated	0	25 Apr 2021	0
80	16 Mar 2021	23 Mar 2021	dose 1	Week 1	25 Apr 2021	0
80	23 Mar 2021	30 Mar 2021	dose 1	Week 2	25 Apr 2021	0
80	30 Mar 2021	6 Apr 2021	dose 1	Week 3	25 Apr 2021	0
80	6 Apr 2021	9 Apr 2021	dose 1	Week 4	25 Apr 2021	0
80	9 Apr 2021	16 Apr 2021	dose 2	Week 1	25 Apr 2021	0
80	16 Apr 2021	23 Apr 2021	dose 2	Week 2	25 Apr 2021	0
80	23 Apr 2021	25 Apr 2021	dose 2	Week 3	25 Apr 2021	1
…						
96	1 Jan 2029	8 May 2021	unvaccinated	0	8 May 2021	1

Time intervals used in the statistical analysis for each COVID-19 outcome

We applied different time intervals for different outcomes due to number of cases. Shorter intervals were applied for infection and hospitalization, while longer intervals were applied for ICU admission and death (see below). An incomplete interval (e.g., 1-6 days for weekly interval) was allowed for the final time interval in a sequence.

**COVID-19 outcomes**	**Time intervals**	**Week no.**
Infection and hospitalization	Weekly	Week 1, 2, 3,4
	Two weeks interval	Week 5-6, 7-8, 9-10
	Three weeks interval	Week 11-13
	Four weeks interval	Week 14-17, 18-21, …, 54-57
ICU admission and death	Weekly	Week 1,2,3
	Two weeks interval	Week 4-5
	Four weeks interval	Week 6-9, 10-13
	Eight weeks interval	Week 14-21, 22-29, …, 46-53, 54-57

Modelling steps in 10% random samples

In the Cox model, each individual's follow-up time was first divided according to vaccination status (unvaccinated, first dose and second dose) and then the vaccination exposure periods further divided into time intervals after each dose, until transition to the next dose. For instance, one individual can be divided into 23 records supposing they had first dose on December 1, 2020, and had second dose after four weeks

and remained in second dose to the end of follow-up (i.e., 1 record for unvaccinated, 4 records for first dose, and 18 records for second dose), such fine division of follow-up time is computationally challenging. Therefore, we performed two pre-analyses in 10% randomly selected individuals to decide the final model used in the full-scale analyses.

One analysis aimed to select appropriate covariates for confounding adjustments, based on change in effect estimate criteria. A list of potential confounders and/or risk factors for COVID-19 were selected based on previous publications, including age, sex, ethnicity, birth country, socioeconomic status, geographic location, cardiovascular diseases, respiratory diseases, kidney function, autoimmune diseases, psychiatric conditions, dementia, cancer, etc. Age and sex were predetermined and always included in the model. Then the other potential confounders and/or risk factors were further included in the model. Inclusion of the covariates that changed effect estimates by >5% were remained in the model. Though ethnicity and geographic location were showed to be important confounders in other studies, they did not meet the criteria of covariates selection (change estimates by >5%), thus were not considered in the final model. eTable 1 shows the difference in HRs from different models and fully adjusted model was used in the full-scale analyses.

Another analysis aimed to confirm if the analysis performed in dose 1 and dose 2 separately can yield similar results to an analysis including both doses simultaneously with an interaction term between dose and time interval (dose × interval). The analysis was performed for COVID-19 infection, and similar results were obtained (eTable 2).

Analysis of vaccine effectiveness for first dose

When analysing the VE for one dose on an outcome, subjects were started to be followed since 1 January 2020, with exposure status changing when they got their first vaccination. The end of follow-up occurred at the time of an outcome event or censoring, defined as second dose, emigration, death, or end of follow-up, whichever came first.

Section S4: Results and Discussion on vaccine effectiveness for first dose

Although two doses of vaccine are required for basic vaccination, some persons remain on one dose for different reasons. Therefore, there is still a need to estimate the VE and evaluate time for VE build-up and durability of single-dose vaccine responses. The results of VE for first dose are listed in eTable 3 and 4, as well as illustrated in eFigure 4. As expected, our results showed overall lower VE against infection with only one dose than with two, although the initial ramping-up period for protection seemed relatively short, i.e., the peak VE was reached at week three. Averagely the VE against infection were <50% and protection was lost from week 30.

For severe COVID-19 outcomes, there was a transient decline in VE from week one to week two with an increase again in the following weeks. Overall, the VE rarely reached 80%. The paradoxical high VE immediately after the first dose, followed by a dip and then an expected rise was previously described in other studies, and it was assumed to be attributed to vaccinated patients being less likely to seek care after vaccination, especially for milder COVID-19-type symptoms and COVID-19 exposure (2).

Table S1. Comparison between models without and with adjusting covariates, in 10% randomly selected individuals. Analyses were performed for COVID-19 infection, estimating vaccine effectiveness (VE) after two doses.

	raw model [a)]			Partly adjusted model [b)]			fully adjusted model [c)]		
**time intervals after two doses**	**VE**	**95%CI_left**	**95%CI_right**	**VE**	**95%CI_left**	**95%CI_right**	**VE**	**95%CI_left**	**95%CI_right**
unvaccinated	Ref.	Ref.	Ref.	Ref.	Ref.	Ref.	Ref.	Ref.	Ref.
week 1	50·7	46·5	54·5	43·0	38·2	47·5	47·7	43·3	51·8
week 2	72·1	68·9	75·1	68·3	64·5	71·6	71·0	67·6	74·1
week 3	73·0	69·7	75·9	69·5	65·8	72·8	72·3	69·0	75·3
week 4	72·5	69·3	75·4	69·2	65·6	72·4	72·2	68·9	75·1
week 5-6	58·4	55·9	60·9	54·0	51·1	56·7	58·7	56·1	61·1
week 7-8	51·4	48·7	54·0	46·6	43·6	49·5	52·5	49·8	55·0
week 9-10	43·3	40·2	46·3	38·4	35·0	41·6	45·4	42·4	48·2
week 11-13	41·2	38·5	43·8	38·0	35·2	40·8	44·8	42·2	47·2
week 14-17	9·1	6·5	11·6	8·8	6·1	11·3	17·6	15·3	20·0
week 18-21	-15·9	-18·3	-13·5	-13·2	-15·7	-10·9	-0·4	-2·6	1·8
week 22-25	-22·9	-25·4	-20·3	-25·7	-28·4	-23·0	-10·4	-12·8	-8·0
week 26-29	-9·3	-12·2	-6·5	-28·8	-32·3	-25·3	-12·0	-15·1	-9·0
week 30-33	7·3	3·7	10·8	-18·9	-23·6	-14·3	-2·9	-7·0	1·0
week 34-37	1·2	-6·2	8·2	-32·2	-42·3	-22·9	-13·0	-21·6	-5·0
week 38-41	17·0	5·6	27·1	-15·7	-31·7	-1·6	2·2	-11·3	14·1
week 42-45	2·4	-10·0	13·5	-28·0	-44·4	-13·5	-3·7	-17·0	8·1
week 46-49	-40·9	-56·4	-26·9	-56·7	-74·0	-41·1	-20·8	-34·1	-8·7
week 50-53	-27·1	-60·0	-1·0	-56·0	-96·3	-23·9	1·0	-24·6	21·4

a) Raw model: without any covariates

b) Partly adjusted model: with age (spline term with 4 knots) and sex

c) Fully adjusted model: with age (spline term with 4 knots), sex, country of birth (Sweden/other countries), health care workers (yes/no), income (low, medium, high using terciles of the study populations, unknown), education (primary, secondary, tertiary, unknown), marital status (married, unmarried, unknown), living at special care facilities and/or receiving home care services (yes/no) and prior comorbidities and treatments (yes/no). Prior comorbidities and treatments included cardiovascular diseases, stroke, hypertension, diabetes, obstructive respiratory diseases, chronic kidney diseases, obesity, autoimmune diseases, dementia, psychiatric conditions, and cancer.

Table S2. Analysis performed in dose 1 and dose 2 separately and analysis including two doses simultaneously with an interaction term between dose and time interval. Analyses were performed for COVID-19 infection in 10% randomly selected individuals.

Analysis in dose 1 and dose 2 separately [a)]						Analysis with interaction term (dose × interval) [a)]				
**week after dose 1**	**Hazard ratio**	**95%CI_left**	**95%CI_right**	**P**		**week after dose 1**	**Hazard ratio**	**95%CI_left**	**95%CI_right**	**P**
week 1	0·8	0·7	0·8	<0·001		dose 1#week 1	0·8	0·8	0·9	<0·001
week 2	0·7	0·6	0·7	<0·001		dose 1#week 2	0·7	0·7	0·8	<0·001
week 3	0·4	0·4	0·5	<0·001		dose 1#week 3	0·5	0·4	0·5	<0·001
week 4	0·4	0·4	0·5	<0·001		dose 1#week 4	0·5	0·4	0·5	<0·001
week 5-6	0·5	0·5	0·6	<0·001		dose 1#week 5-6	0·6	0·5	0·6	<0·001
week 7-8	0·7	0·6	0·7	<0·001		dose 1#week 7-8	0·7	0·7	0·8	<0·001
week 9-10	0·6	0·5	0·7	<0·001		dose 1#week 9-10	0·6	0·5	0·7	<0·001
week 11-13	0·7	0·6	0·8	<0·001		dose 1#week 11-13	0·7	0·6	0·8	<0·001
week 14-17	0·8	0·7	1·0	0·01		dose 1#week 14-17	0·8	0·7	0·9	<0·001
week 18-21	0·9	0·7	1·0	0·02		dose 1#week 18-21	0·7	0·6	0·8	<0·001
week 22-25	1·1	0·9	1·2	0·43		dose 1#week 22-25	0·9	0·8	1·0	0·19
week 26-29	1·1	0·9	1·2	0·45		dose 1#week 26-29	0·9	0·8	1·1	0·19
week 30-33	0·9	0·7	1·1	0·39		dose 1#week 30-33	0·8	0·6	1·0	0·03
week 34-37	0·8	0·6	1·1	0·27		dose 1#week 34-37	0·7	0·5	1·0	0·03
week 38-41	0·6	0·4	0·9	0·03		dose 1#week 38-41	0·5	0·3	0·8	0·01
week 42-45	1·0	0·6	1·5	0·92		dose 1#week 42-45	0·8	0·5	1·2	0·32
week 46-49	1·2	0·8	1·7	0·37		dose 1#week 46-49	1·0	0·6	1·4	0·80
week 50-53	1·4	0·8	2·5	0·25		dose 1#week 50-53	1·1	0·6	2·0	0·75
week 54-57	0·7	0·1	5·0	0·73		dose 1#week 54-57	0·6	0·1	4·1	0·58
**week after dose 2**	**Hazard ratio**	**95%CI_left**	**95%CI_right**	**P**		**week after dose 2**	**Hazard ratio**	**95%CI_left**	**95%CI_right**	**P**
week 1	0·5	0·5	0·5	<0·001		dose 2#week 1	0·5	0·4	0·5	<0·001
week 2	0·3	0·2	0·3	<0·001		dose 2#week 2	0·3	0·2	0·3	<0·001
week 3	0·3	0·2	0·3	<0·001		dose 2#week 3	0·3	0·2	0·3	<0·001
week 4	0·3	0·2	0·3	<0·001		dose 2#week 4	0·3	0·2	0·3	<0·001
week 5-6	0·4	0·4	0·4	<0·001		dose 2#week 5-6	0·4	0·4	0·4	<0·001
week 7-8	0·5	0·5	0·5	<0·001		dose 2#week 7-8	0·5	0·4	0·5	<0·001

week 9-10	0·6	0·5	0·6	<0·001		dose 2#week 9-10	0·5	0·5	0·6	<0·001
week 11-13	0·6	0·6	0·6	<0·001		dose 2#week 11-13	0·6	0·5	0·6	<0·001
week 14-17	0·9	0·9	0·9	<0·001		dose 2#week 14-17	0·9	0·8	0·9	<0·001
week 18-21	1·2	1·1	1·2	<0·001		dose 2#week 18-21	1·1	1·1	1·1	<0·001
week 22-25	1·2	1·2	1·3	<0·001		dose 2#week 22-25	1·2	1·2	1·2	<0·001
week 26-29	1·1	1·1	1·1	<0·001		dose 2#week 26-29	1·0	1·0	1·1	<0·001
week 30-33	0·9	0·9	1·0	<0·001		dose 2#week 30-33	0·9	0·9	0·9	<0·001
week 34-37	1·0	0·9	1·1	0·74		dose 2#week 34-37	0·9	0·9	1·0	0·14
week 38-41	0·8	0·7	0·9	0·01		dose 2#week 38-41	0·8	0·7	0·9	<0·001
week 42-45	1·0	0·9	1·1	0·69		dose 2#week 42-45	0·9	0·8	1·1	0·29
week 46-49	1·4	1·3	1·6	<0·001		dose 2#week 46-49	1·4	1·2	1·5	<0·001
week 50-53	1·3	1·0	1·6	0·04		dose 2#week 50-53	1·2	1·0	1·5	0·09

a) Model was run without any covariates (raw model).

Table S3. Vaccine effectiveness (VE) against COVID-19 infection and hospitalization per time interval after one dose

	COVID-19 infection					COVID-19 hospitalization				
**time intervals after one dose**	**no**	**yes**	**total**	**VE (95%CI)**	**P**	**no**	**yes**	**total**	**VE (95%CI)**	**P**
unvaccinated	8005887	1147569	9153456	Ref	Ref	9083783	69673	9153456	Ref	Ref
week 1	6620724	5665	6626389	9·9 (8·4, 11·5)	<0·001	7439960	168	7440128	65·6 (62·5, 68·5)	<0·001
week 2	6597474	3179	6600653	18·6 (17·1, 20·1)	<0·001	7412903	156	7413059	22·1 (17·2, 26·6)	<0·001
week 3	6561187	3028	6564215	49·8 (48·6, 51)	<0·001	7368845	88	7368933	44 (39·8, 47·9)	<0·001
week 4	6517229	3516	6520745	49·1 (47·8, 50·3)	<0·001	7314900	93	7314993	67·7 (64·1, 71)	<0·001
week 5-6	6485350	10998	6496348	43·7 (42·7, 44·8)	<0·001	7286468	164	7286632	76·6 (73·8, 79)	<0·001
week 7-8	6435795	14527	6450322	34·1 (32·5, 35·7)	<0·001	7239871	186	7240057	73 (67·7, 77·4)	<0·001
week 9-10	6318747	14800	6333547	36·6 (34·2, 38·8)	<0·001	7104688	171	7104859	71·8 (63·9, 77·9)	<0·001
week 11-13	6183339	21360	6204699	38 (35·3, 40·6)	<0·001	6954370	266	6954636	72·5 (62·5, 79·9)	<0·001
week 14-17	6001324	63727	6065051	22·9 (19·6, 26)	<0·001	6798447	405	6798852	71·7 (59·2, 80·3)	<0·001
week 18-21	5659213	163935	5823148	15·8 (12·4, 19·2)	<0·001	6554252	583	6554835	67 (51·1, 77·7)	<0·001
week 22-25	5008930	170797	5179727	9 (4·9, 13)	<0·001	5887753	691	5888444	60·9 (42·1, 73·6)	<0·001
week 26-29	3250490	81750	3332240	7·3 (2·5, 11·9)	<0·001	3743234	838	3744072	68·5 (50·5, 79·9)	<0·001
week 30-33	1291929	30211	1322140	-3·4 (-10·3, 3)	0·30	1500226	630	1500856	54·4 (31·9, 69·4)	<0·001
week 34-37	528513	7352	535865	14 (5·7, 21·5)	<0·001	602531	293	602824	40 (9·6, 60·2)	0·02
week 38-41	263244	2434	265678	22·1 (10·6, 32·2)	<0·001	296221	193	296414	3 (-46·1, 35·6)	0·88
week 42-45	121552	2766	124318	-11·8 (-26·5, 1·1)	0·08	141173	155	141328	18·4 (-40·7, 52·7)	0·47
week 46-49	36665	3640	40305	-35·6 (-51, -21·9)	<0·001	48851	79	48930	0·4 (-75·5, 43·5)	0·99
week 50-53	11461	889	12350	-27·6 (-53·2, -6·3)	0·01	17320	21	17341	-6·7 (-137·7, 52·1)	0·87
week 54-57	203	5	208	11·5 (-38·7, 43·6)	0·59	327	0	327		

Table S4. Vaccine effectiveness (VE) against COVID-19 ICU admission and death per time interval after one dose

	COVID-19 ICU admission					COVID-19 related death				
**time intervals after one dose**	**no**	**yes**	**total**	**VE (95%CI)**	**P**	**no**	**yes**	**total**	**VE (95%CI)**	**P**
unvaccinated	9145795	7661	9153456	Ref	Ref	9138405	15051	9153456	Ref	Ref
week 1	7335788	2	7335790	86·5 (80·1, 90·8)	<0·001	7488449	37	7488486	88·7 (85·4, 91·3)	<0·001
week 2	7318097	6	7318103	45·9 (33·7, 55·9)	<0·001	7461306	40	7461346	64·6 (58·6, 69·7)	<0·001
week 3	7276817	7	7276824	54 (42·2, 63·3)	<0·001	7417043	32	7417075	45·9 (38, 52·7)	<0·001
week 4-5	7213798	8	7213806	76·6 (69·6, 82·1)	<0·001	7362785	48	7362833	12·8 (1, 23·1)	0·03
week 6-9	7066867	11	7066878	82·5 (74·5, 88)	<0·001	7315102	98	7315200	28·6 (13·7, 40·9)	<0·001
week 10-13	6841443	14	6841457	84·3 (58·1, 94·1)	<0·001	7078937	80	7079017	44·7 (18·8, 62·3)	<0·001
week 14-21	6592925	36	6592961	93·2 (51·7, 99)	0·01	6843980	111	6844091	67·9 (46·5, 80·8)	<0·001
week 22-29	4475859	60	4475919	89·7 (26·8, 98·6)	0·02	5927820	142	5927962	64·3 (24·3, 83·1)	0·01
week 30-37	1055120	26	1055146	45·2 (-70·7, 82·4)	0·30	1516278	247	1516525	38·4 (-8·2, 64·9)	0·09
week 38-45	279478	0	279478			299652	82	299734	12·9 (-69·2, 55·1)	0·68
week 46-53	27850	0	27850			49464	24	49488	42·6 (-53·9, 78·6)	0·27
week 54-57	na [a)]	na	na			333	0	333		

a) na, not applicable. For ICU admission, the end of follow-up was on 31 Dec 2021 due to data availability.

Table S5. Intervals between vaccine doses among individuals who received at least two doses of vaccination.

Interval between doses (weeks)		Mean	p5	p25	p50	p75	p95
All	dose 1 to 2	7	3	6	6	7	11
	dose 2 to 3	28	22	25	27	29	38
Homologous BNT162b2	dose 1 to 2	6	3	5	6	7	8
	dose 2 to 3	28	22	25	27	30	39
Homologous mRNA-1273	dose 1 to 2	6	4	5	6	7	8
	dose 2 to 3	28	22	25	27	30	36
homologous AZD1222	dose 1 to 2	10	9	9	10	10	12
	dose 2 to 3	25	22	23	24	26	31

Table S6. Vaccine effectiveness (VE) against COVID-19 infection and hospitalization per time interval after two doses

	COVID-19 infection					COVID-19 hospitalization				
**time intervals after two doses**	**no**	**yes**	**total**	**VE (95%CI)**	**P**	**no**	**yes**	**total**	**VE (95%CI)**	**P**
unvaccinated	8005887	1147569	9153456	Ref	Ref	9083783	69673	9153456	Ref	Ref
week 1	6620724	5665	6626389	50·2 (48·8, 51·5)	<0·001	7439960	168	7440128	82·7 (79·9, 85·2)	<0·001
week 2	6597474	3179	6600653	71·1 (70·0, 72·1)	<0·001	7412903	156	7413059	83·4 (80·5, 85·8)	<0·001
week 3	6561187	3028	6564215	72·0 (71·0, 73·0)	<0·001	7368845	88	7368933	90·4 (88·2, 92·2)	<0·001
week 4	6517229	3516	6520745	69·1 (68·1, 70·1)	<0·001	7314900	93	7314993	89·7 (87·4, 91·6)	<0·001
week 5-6	6485350	10998	6496348	58·7 (57·9, 59·5)	<0·001	7286468	164	7286632	90·9 (89·4, 92·2)	<0·001
week 7-8	6435795	14527	6450322	49·2 (48·4, 50·1)	<0·001	7239871	186	7240057	89·6 (88·0, 91·0)	<0·001
week 9-10	6318747	14800	6333547	43·9 (42·9, 44·8)	<0·001	7104688	171	7104859	90·4 (88·8, 91·7)	<0·001
week 11-13	6183339	21360	6204699	43·9 (43·1, 44·6)	<0·001	6954370	266	6954636	89·9 (88·6, 91·1)	<0·001
week 14-17	6001324	63727	6065051	19·5 (18·8, 20·2)	<0·001	6798447	405	6798852	87·5 (86·2, 88·7)	<0·001
week 18-21	5659213	163935	5823148	-1·2 (-1·9, -0·5)	<0·001	6554252	583	6554835	83·8 (82·4, 85·2)	<0·001
week 22-25	5008930	170797	5179727	-10·9 (-11·7, -10·2)	<0·001	5887753	691	5888444	83·8 (82·5, 85·0)	<0·001
week 26-29	3250490	81750	3332240	-12·8 (-13·7, -11·8)	<0·001	3743234	838	3744072	74·4 (72·4, 76·2)	<0·001
week 30-33	1291929	30211	1322140	-0·4 (-1·7, 0·8)	0·52	1500226	630	1500856	64·1 (60·9, 66·9)	<0·001
week 34-37	528513	7352	535865	-10·8 (-13·4, -8·2)	<0·001	602531	293	602824	53·5 (47·6, 58·7)	<0·001
week 38-41	263244	2434	265678	-2·4 (-6·6, 1·6)	0·24	296221	193	296414	34·9 (24·8, 43·7)	<0·001
week 42-45	121552	2766	124318	-5·0 (-9·0, -1·1)	0·01	141173	155	141328	32·5 (20·7, 42·5)	<0·001
week 46-49	36665	3640	40305	-22·9 (-27·0, -18·9)	<0·001	48851	79	48930	39·3 (24·2, 51·5)	<0·001
week 50-53	11461	889	12350	-21·2 (-29·5, -13·5)	<0·001	17320	21	17341	54·8 (30·5, 70·6)	<0·001
week 54-57	203	5	208	8·8 (-119, 62·1)	0·84	327	0	327		

Table S7. Vaccine effectiveness (VE) against COVID-19 ICU admission and death per time interval after two doses

	COVID-19 ICU admission					COVID-19 related death				
**time intervals after two doses**	**no**	**yes**	**total**	**VE (95%CI)**	**P**	**no**	**yes**	**total**	**VE (95%CI)**	**P**
unvaccinated	9145795	7661	9153456	Ref	Ref	9138405	15051	9153456	Ref	Ref
week 1	7335788	2	7335790	97·9 (91·7, 99·5)	<0·001	7488449	37	7488486	88·8 (84·5, 91·9)	<0·001
week 2	7318097	6	7318103	93·3 (85·0, 97·0)	<0·001	7461306	40	7461346	86·8 (81·9, 90·3)	<0·001
week 3	7276817	7	7276824	91·9 (82·9, 96·1)	<0·001	7417043	32	7417075	88·8 (84·1, 92·1)	<0·001
week 4-5	7213798	8	7213806	95·2 (90·4, 97·6)	<0·001	7362785	48	7362833	91·7 (89·0, 93·8)	<0·001
week 6-9	7066867	11	7066878	97·0 (94·6, 98·4)	<0·001	7315102	98	7315200	91·7 (89·7, 93·2)	<0·001
week 10-13	6841443	14	6841457	97·0 (94·9, 98·2)	<0·001	7078937	80	7079017	93·6 (91·9, 94·9)	<0·001
week 14-21	6592925	36	6592961	95·3 (93·4, 96·7)	<0·001	6843980	111	6844091	92·9 (91·2, 94·2)	<0·001
week 22-29	4475859	60	4475919	91·2 (88·4, 93·3)	<0·001	5927820	142	5927962	84·2 (80·8, 87·0)	<0·001
week 30-37	1055120	26	1055146	73·0 (59·7, 81·9)	<0·001	1516278	247	1516525	63·6 (56·8, 69·3)	<0·001
week 38-45	279476	2	279478	91·6 (66·4, 97·9)	<0·001	299652	82	299734	43·6 (28·5, 55·5)	<0·001
week 46-53	27850	0	27850			49464	24	49488	69·6 (54·1, 79·9)	<0·001
week 54-57	na [a)]	na	na			333	0	333		

a) na, not applicable. For ICU admission, the end of follow-up was on 31 Dec 2021 due to data availability.

Table S8. Vaccine effectiveness (VE) against COVID-19 infection during pre-Omicron and Omicron period, per time interval after two doses

	Pre-Omicron period					Omicron period [a)]				
**time intervals after two doses**	**no**	**yes**	**total**	**VE (95%CI)**	**P**	**no**	**yes**	**total**	**VE (95%CI)**	**P**
unvaccinated	8005887	1147569	9153456	Ref	Ref	9026457	126999	9153456	ref	ref
week 1	6315590	2360	6317950	85 (84·4, 85·6)	<0·001	6623068	3321	6626389	75·1 (74·3, 76)	<0·001
week 2	6251322	1004	6252326	93·3 (92·9, 93·7)	<0·001	6598470	2183	6600653	45·3 (43, 47·6)	<0·001
week 3	6193294	773	6194067	94·5 (94·1, 94·9)	<0·001	6561959	2256	6564215	44·4 (42, 46·7)	<0·001
week 4	6144240	845	6145085	93·4 (92·9, 93·8)	<0·001	6518072	2673	6520745	43 (40·8, 45·2)	<0·001
week 5-6	6112538	2120	6114658	92·4 (92·1, 92·7)	<0·001	6487443	8905	6496348	31·1 (29·6, 32·6)	<0·001
week 7-8	6032675	2628	6035303	91·8 (91·5, 92·2)	<0·001	6438362	11960	6450322	18·1 (16·6, 19·7)	<0·001
week 9-10	5921110	3237	5924347	92·2 (91·9, 92·5)	<0·001	6321883	11664	6333547	6·4 (4·6, 8·2)	<0·001
week 11-13	5726040	6021	5732061	93·4 (93·3, 93·6)	<0·001	6188753	15946	6204699	11·1 (9·6, 12·5)	<0·001
week 14-17	5162308	13980	5176288	91·4 (91·3, 91·6)	<0·001	6008966	56085	6065051	-2·7 (-3·7, -1·6)	<0·001
week 18-21	3937940	10657	3948597	88·5 (88·3, 88·7)	<0·001	5664953	158195	5823148	-18·9 (-19·9, -18)	<0·001
week 22-25	3179910	10242	3190152	91·4 (91·3, 91·6)	<0·001	5014390	165337	5179727	-30·4 (-31·5, -29·4)	<0·001
week 26-29	1632969	4484	1637453	91 (90·8, 91·3)	<0·001	3253132	79108	3332240	-42·7 (-44·2, -41·3)	<0·001
week 30-33	657862	1499	659361	84·3 (83·5, 85·1)	<0·001	1293171	28969	1322140	-34·2 (-36, -32·4)	<0·001
week 34-37	398006	1071	399077	85·2 (84·3, 86·1)	<0·001	529442	6423	535865	-53·4 (-57·3, -49·5)	<0·001
week 38-41	211322	955	212277	90·9 (90·3, 91·5)	<0·001	263987	1691	265678	-52·5 (-60·1, -45·3)	<0·001
week 42-45	51113	412	51525	92·4 (91·6, 93·1)	<0·001	121693	2625	124318	-50·6 (-56·6, -44·8)	<0·001
week 46-49	57	0	57			36665	3640	40305	-65·3 (-70·8, -59·9)	<0·001
week 50-53						11461	889	12350	-95·7 (-109, -83·1)	<0·001
week 54-57						203	5	208		

a) The analysis of VE during Omicron period was modelled the entire follow-up period but only events after December 1, 2021, were considered as incident cases for estimation, and individuals with events before that were censored at their event.

Table S9. Vaccine effectiveness (VE) against COVID-19 hospitalization during pre-Omicron and Omicron period, per time interval after two doses

	Pre-Omicron period					Omicron period [a)]				
time intervals after two doses	no	yes	total	VE (95%CI)	P	no	yes	total	VE (95%CI)	P
unvaccinated	9083783	69673	9153456	Ref	Ref	9150213	3243	9153456	ref	ref
week 1	7059367	149	7059516	85·8 (83·3, 87·9)	<0·001	7440109	19	7440128	90·5 (85·1, 94)	<0·001
week 2	6984334	141	6984475	86·1 (83·6, 88·2)	<0·001	7413044	15	7413059	75·6 (59·5, 85·3)	<0·001
week 3	6917209	86	6917295	91·4 (89·4, 93·1)	<0·001	7368931	2	7368933	96·8 (87·1, 99·2)	<0·001
week 4	6860865	87	6860952	91·2 (89·1, 92·9)	<0·001	7314987	6	7314993	90·6 (79·1, 95·8)	<0·001
week 5-6	6825157	142	6825299	93 (91·7, 94·1)	<0·001	7286610	22	7286632	86·3 (79·2, 91)	<0·001
week 7-8	6730867	145	6731012	93 (91·7, 94)	<0·001	7240015	42	7240057	78·1 (70·3, 83·9)	<0·001
week 9-10	6598086	124	6598210	94·2 (93·1, 95·1)	<0·001	7104811	48	7104859	77 (69·4, 82·7)	<0·001
week 11-13	6367628	181	6367809	94·8 (94, 95·5)	<0·001	6954546	90	6954636	76·8 (71·3, 81·2)	<0·001
week 14-17	5710313	225	5710538	95·5 (94·8, 96)	<0·001	6798657	195	6798852	79 (75·6, 81·8)	<0·001
week 18-21	4281641	249	4281890	94·4 (93·6, 95)	<0·001	6554483	352	6554835	79·9 (77·5, 82·1)	<0·001
week 22-25	3422214	336	3422550	95·1 (94·5, 95·6)	<0·001	5888013	431	5888444	81 (78·9, 82·9)	<0·001
week 26-29	1734812	339	1735151	91·2 (90·1, 92·1)	<0·001	3743495	577	3744072	71 (68·1, 73·6)	<0·001
week 30-33	704510	185	704695	85·7 (83·5, 87·7)	<0·001	1500394	462	1500856	59·2 (54·8, 63·1)	<0·001
week 34-37	434908	120	435028	87·5 (85, 89·6)	<0·001	602630	194	602824	51·4 (43·6, 58·1)	<0·001
week 38-41	236296	42	236338	91·8 (88·8, 93·9)	<0·001	296253	161	296414	34 (22·3, 44)	<0·001
week 42-45	58570	10	58580	95·3 (91·2, 97·5)	<0·001	141175	153	141328	36·4 (24·8, 46·3)	<0·001
week 46-49	67	0	67			48851	79	48930	40·3 (25, 52·5)	<0·001
week 50-53						17320	21	17341	53 (27·1, 69·7)	<0·001
week 54-57						327	0	327		

a) The analysis of VE during Omicron period was modelled the entire follow-up period but only events after December 1, 2021, were considered as incident cases for estimation, and individuals with events before that were censored at their event.

Figure S1. Trend of COVID-19 infection (a) and hospitalization (b) from January 1, 2020 to January 31, 2022

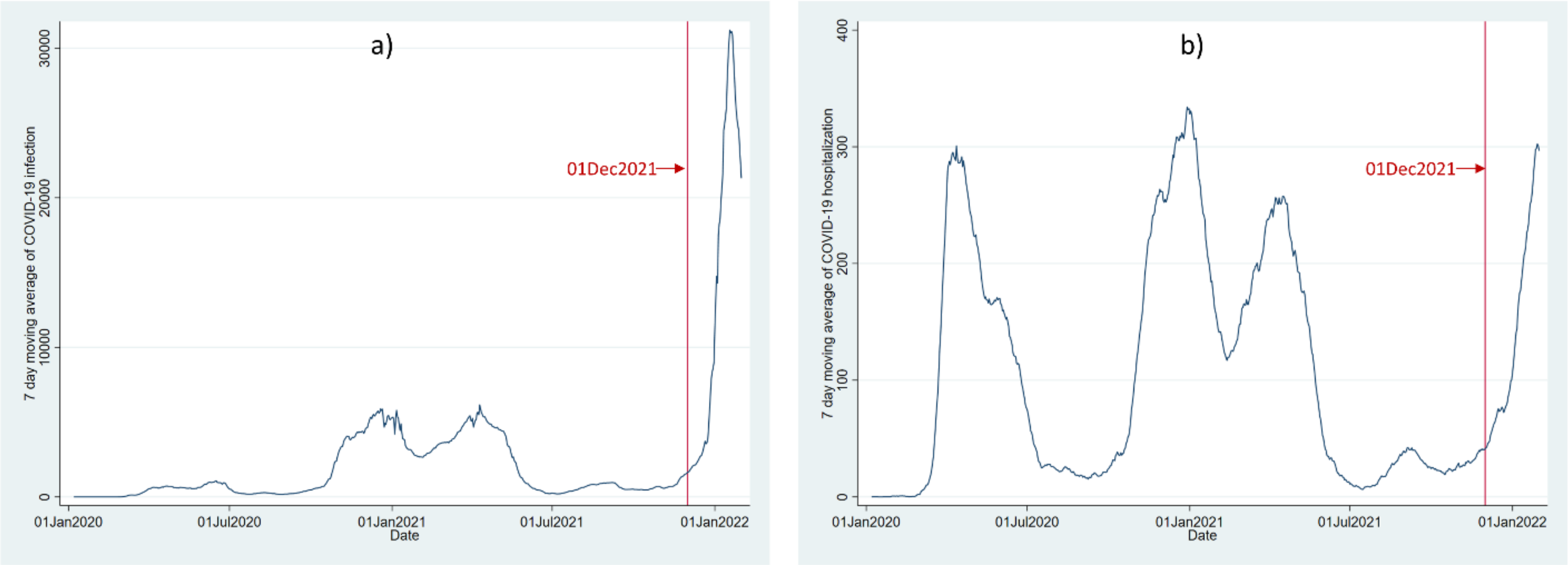

Figure legend: 7-day moving average of cases of COVID-19 infection (a) and hospitalization (b) during the follow-up. Red lines indicate December 21, 2021, the start date of Omicron period used in the study. There was an obvious increase in infection cases after December 21, 2021.

Figure S2. Cumulative proportion of vaccine uptake over time among whole Swedish population (a) and 65+ (b)

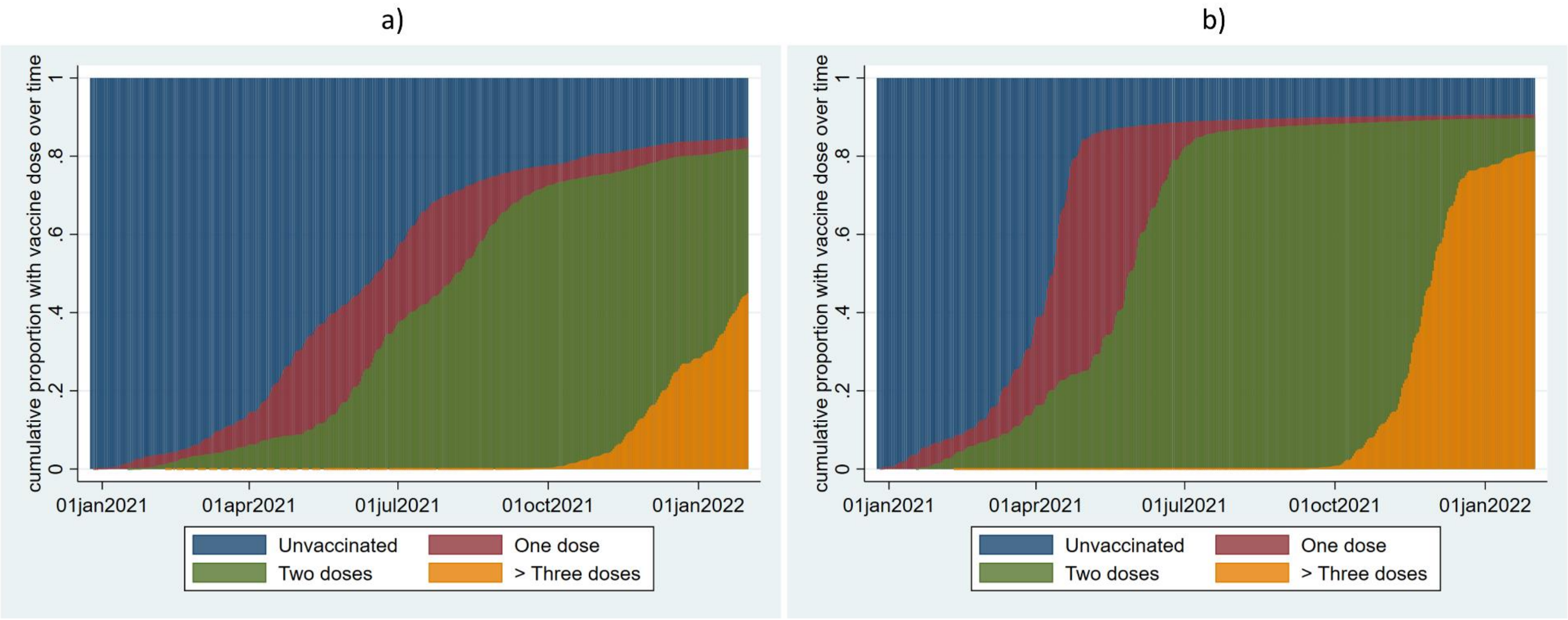

Figure S3. Cumulative count of homologous vaccine type over time

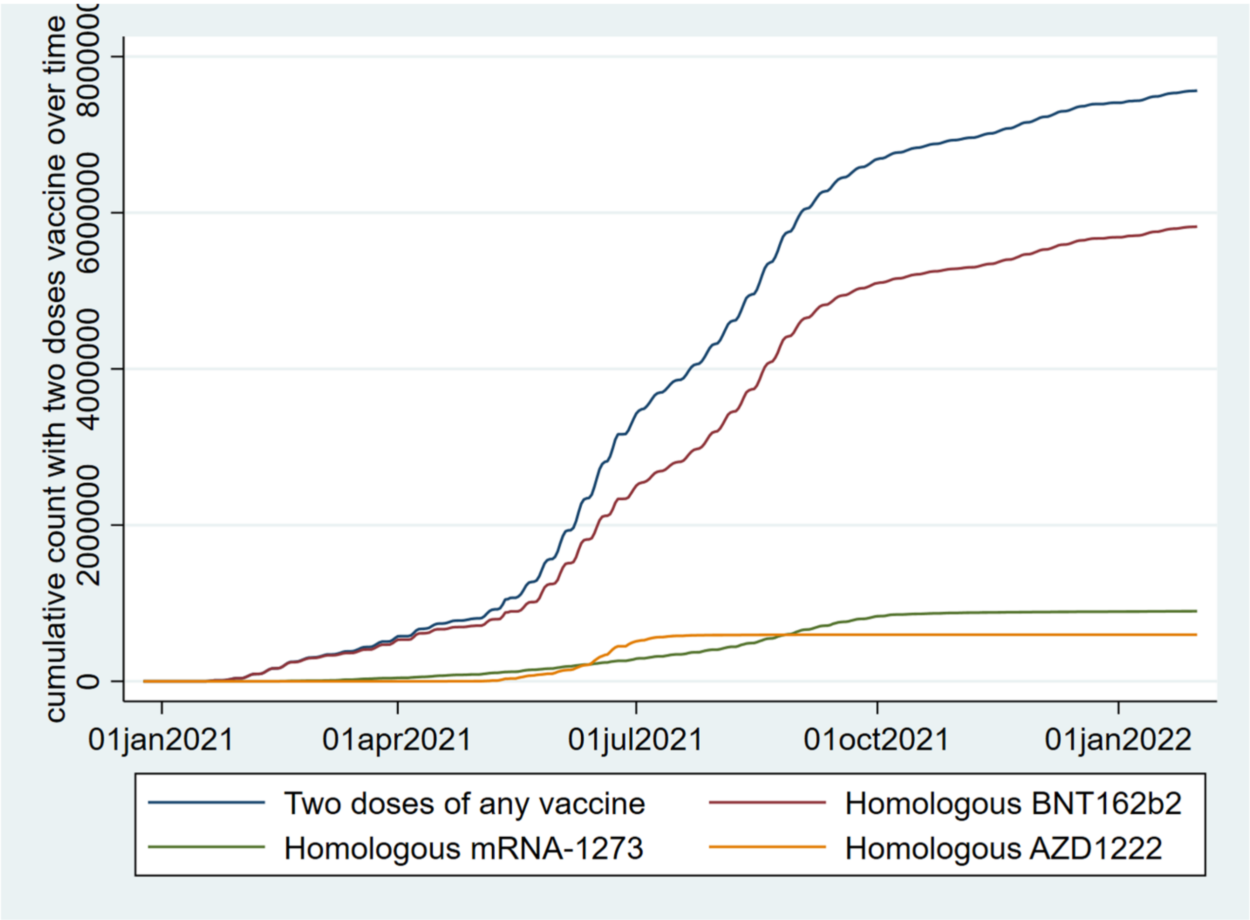

Figure legend: BNT162b2 is the dominate vaccine brand used in Sweden. AZD1222 was stopped from July 2021.

Figure S4. Vaccine effectiveness against COVID-19 infection (a), hospitalization (b) and severe outcomes [ICU admission (c), death (d)] after one dose.

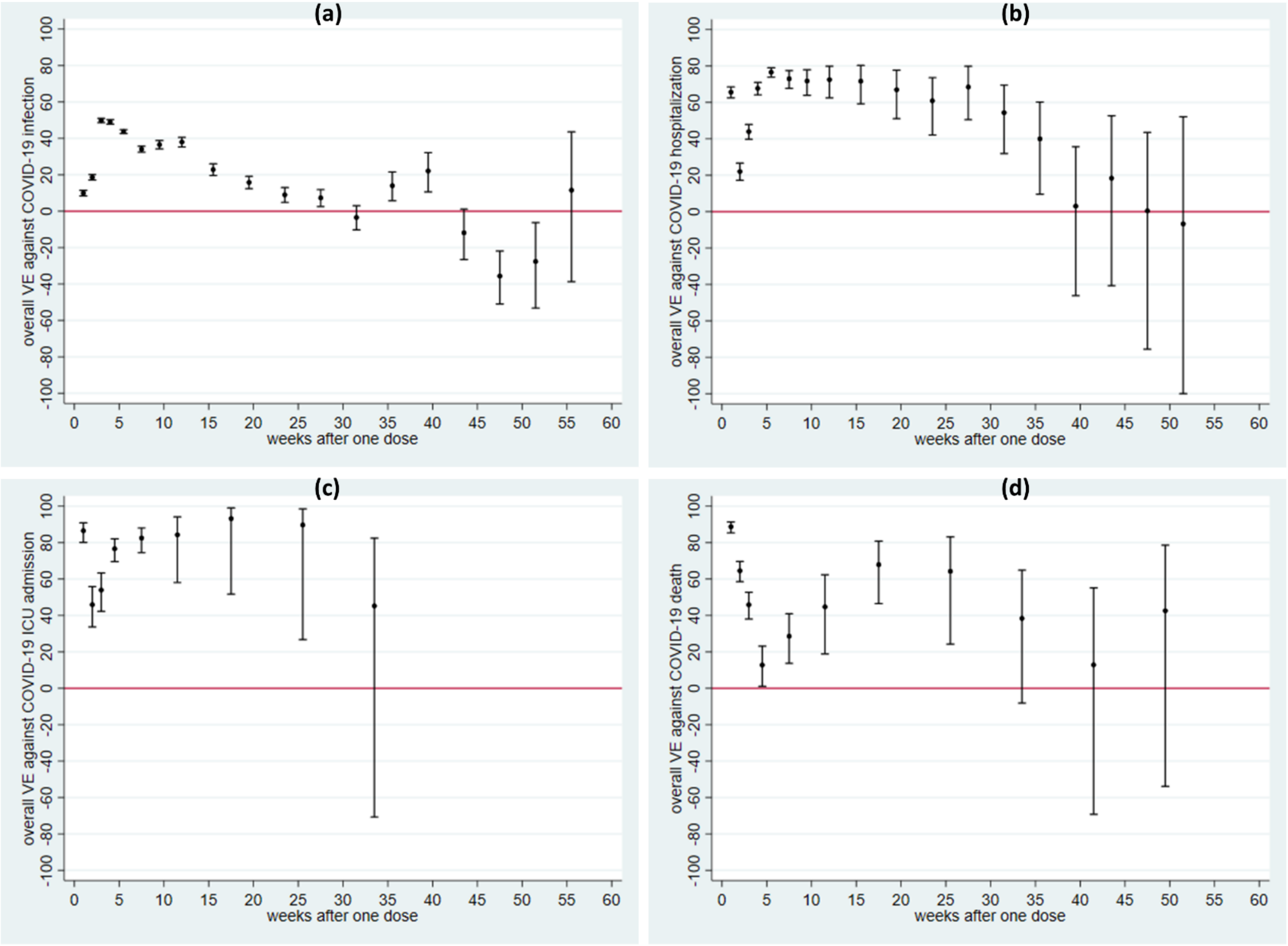

Figure legend: VE denotes vaccine effectiveness. Dots are estimated vaccine effectiveness (dots) and capped spikes are 95% confidence intervals. Red line indicate VE=0.

Figure S5. Vaccine effectiveness against COVID-19 infection (a, c) and hospitalization (b, d) after two doses in each age group

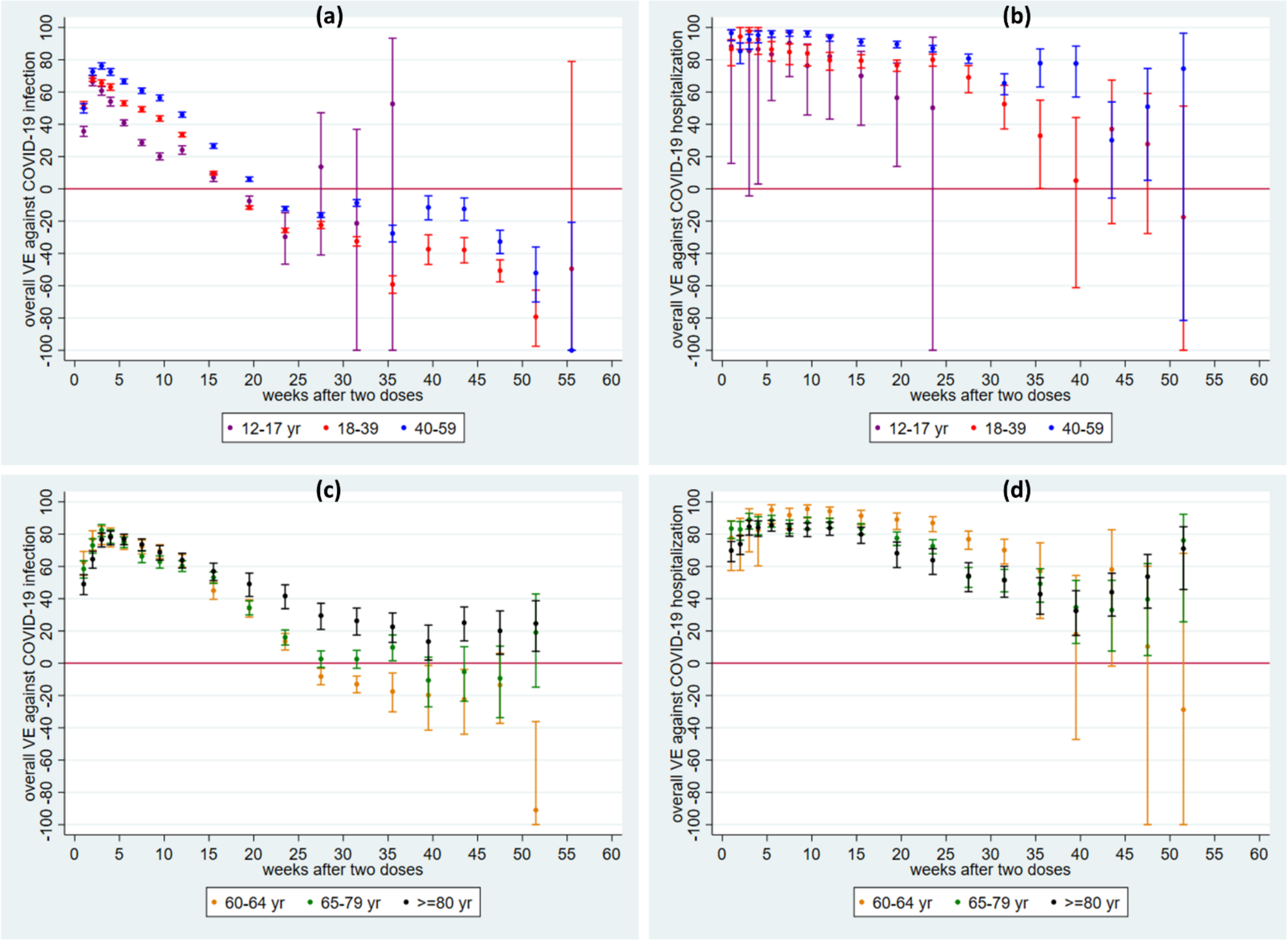

Figure Legend: VE denotes vaccine effectiveness. Dots are estimated vaccine effectiveness (dots) and capped spikes are 95% confidence intervals. Red horizontal line indicate VE=0.

Figure S6. Overall vaccine effectiveness against COVID-19 infection (a) and hospitalization (b) after two doses in each sex stratum

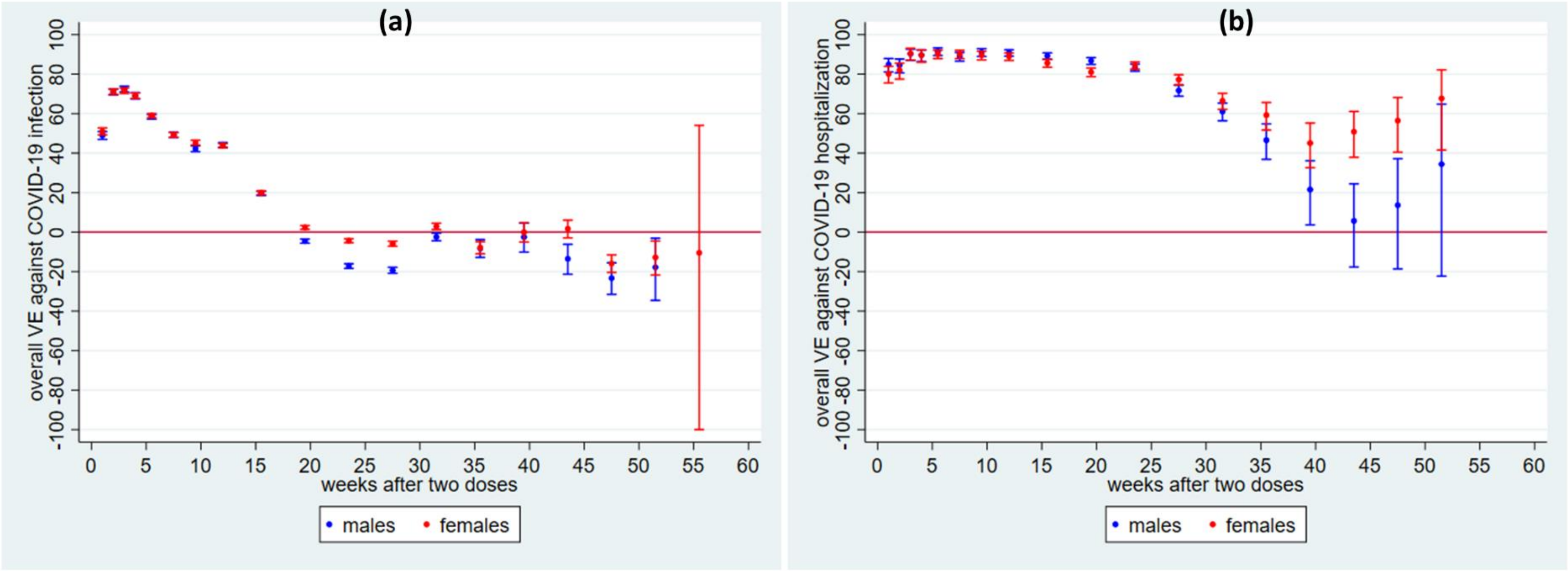

Figure legend: VE denotes vaccine effectiveness. Dots are estimated vaccine effectiveness (dots) and capped spikes are 95% confidence intervals. Red horizontal line indicate VE=0.

Figure S7. Overall vaccine effectiveness against COVID-19 infection (a) and hospitalization (b) by different homologous vaccine type among individuals above age 65.

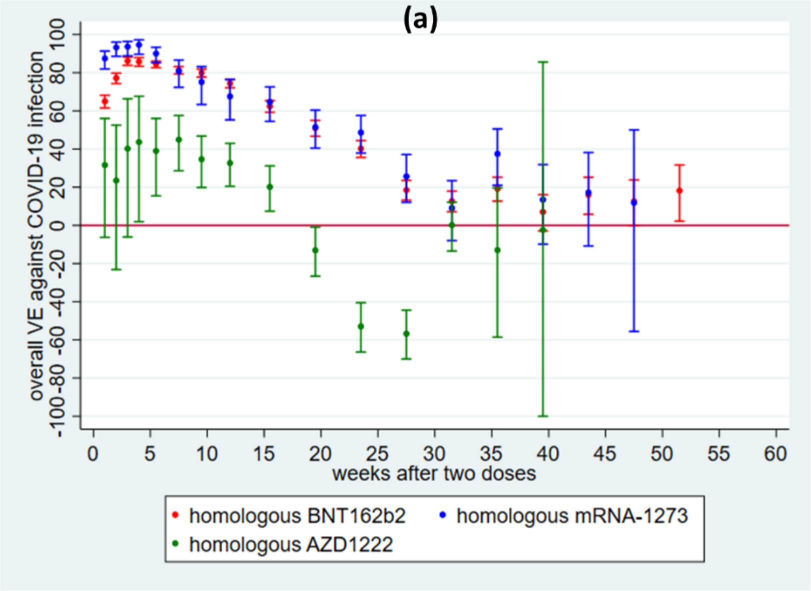

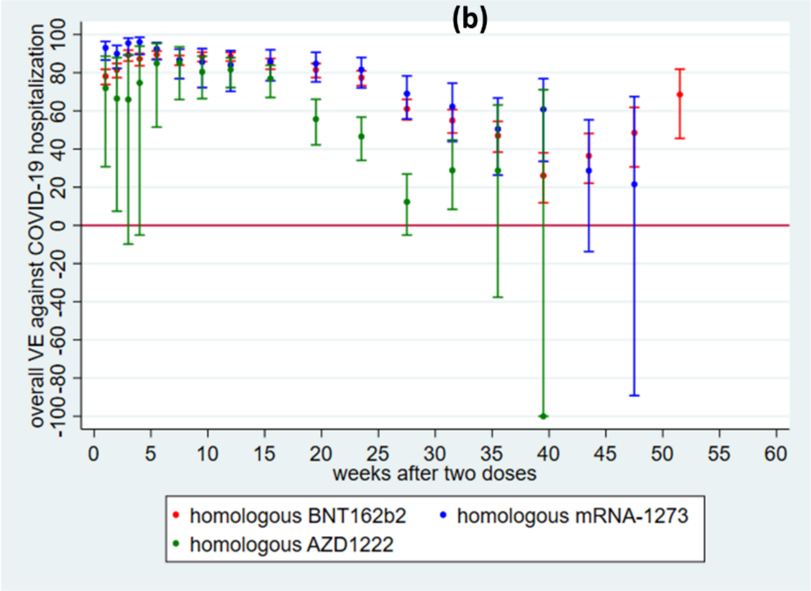

Figure legend: VE denotes vaccine effectiveness. Dots are estimated vaccine effectiveness (dots) and capped spikes are 95% confidence intervals. Red horizontal line indicate VE=0.

References:

1. Nyberg F, Franzén S, Lindh M, et al. Swedish Covid-19 Investigation for Future Insights - A Population Epidemiology Approach Using Register Linkage (SCIFI-PEARL). Clin Epidemiol 2021;13:649–59.

2. Ostropolets A, Hripcsak G. COVID-19 vaccination effectiveness rates by week and sources of bias: a retrospective cohort study. BMJ open. 2022, 12, e061126.
